# Electroencephalographic and early communicative abnormalities in Brattleboro rats

**DOI:** 10.1002/phy2.100

**Published:** 2013-10-20

**Authors:** Robert E Lin, Lauren Ambler, Eddie N Billingslea, Jimmy Suh, Shweta Batheja, Valerie Tatard-Leitman, Robert E Featherstone, Steven J Siegel

**Affiliations:** Department of Psychiatry, Translational Neuroscience Program, School of Medicine, University of PennsylvaniaPhiladelphia, Pennsylvania

**Keywords:** Brattleboro, ERP, schizophrenia, USV, vasopressin

## Abstract

Reductions in the levels of the neuropeptide vasopressin (VP) and its receptors have been associated with schizophrenia. VP is also critical for appropriate social behaviors in humans as well as rodents. One of the prominent symptoms of schizophrenia is asociality and these symptoms may develop prodromally. A reduction in event-related potential (ERP) peak amplitudes is an endophenotype of schizophrenia. In this study, we use the Brattleboro (BRAT) rat to assess the role of VP deficiency in vocal communication during early development and on auditory ERPs during adulthood. BRAT rats had similar vocal communication to wild-type littermate controls during postnatal days 2 and 5 but the time between vocalizations was increased and the power of the vocalizations was reduced beginning at postnatal day 9. During adulthood, BRAT rats had deficits in auditory ERPs including reduced N40 amplitude and reduced low and high gamma intertrial coherence. These results suggest that the role of VP on vocal communication is an age-dependent process. Additionally, the deficits in ERPs indicate an impairment of auditory information processing related to the reduction in VP. Therefore, manipulation of the VP system could provide a novel mechanism for treatment for negative symptoms of schizophrenia.

## Introduction

Auditory event-related potentials (ERPs) are measures of brain electrical activity that are time locked to an auditory stimulus. Schizophrenia patients have abnormal ERP responses to auditory stimuli, which have led to ERPs being proposed as a biomarker for schizophrenia (Luck et al. 2011; Gandal et al. 2012). For instance, previous clinical studies using EEG have shown elevated baseline gamma activity at both frontal and total (e.g., all combined) electrode sites in patients with schizophrenia (Hamm et al. 2012). In addition to the surface ERP and EEG recorded in humans, similar techniques may be used to record from specific intracranial regions in rodents. The resulting rodent ERP and EEG produce an analogous response to humans, allowing for a high degree of translatability (Amann et al. 2008; Luck et al. 2011). Consistent with the human condition, mouse models of schizophrenia have demonstrated increased baseline gamma activity prior to auditory stimuli when using low impedance differential electrodes across multiple brain regions (Ehrlichman et al. 2009; Gandal et al. 2012; Hamm et al. 2012; Saunders et al. 2012a,b). Additionally, schizophrenia patients have a reduction in the N100 peak amplitude of the ERP which is replicated as an N40 amplitude reduction in schizophrenia rodent models (Maxwell et al. 2004; Halene et al. 2009; Amann et al. 2008; Dias et al. 2011; Featherstone et al. 2012). Previous studies in humans have shown that vasopressin (VP) enhances ERP peaks during an oddball paradigm (Born et al. 1998). Additionally, a VP agonist (DDAVP) given to schizophrenia patients improves negative symptom profiles. However, others have also shown no effect on memory impairments (Blake et al. 1978; Jenkins et al. 1979; Hostetter et al. 1980; Sahgal et al. 1982; Stein et al. 1984; Brambilla et al. 1986, 1989). Gene association studies have indicated a link between the promoter region of the V1aR gene and prepulse inhibition, an endophenotype of schizophrenia (Levin et al. 2009). Moreover, there is a reduction in circulating and CNS VP in schizophrenia, but several studies show no change. (Linkowski et al. 1984; Gjerris et al. 1985; Sorensen et al. 1985; Kishimoto et al. 1989; Legros and Ansseau 1992; Legros et al. 1992; Krishnamurthy et al. 2012; Elman et al. 2003). Furthermore, apomorphine-induced stimulation of VP levels in blood is significantly blunted in schizophrenia patients (Legros et al. 1992). Based on these studies VP is likely to be at least partially involved in the pathophysiology of schizophrenia.

The neurodevelopmental pathophysiology of schizophrenia produces prodromal symptoms prior to the onset of disease (MacCabe et al. 2013; Oribe et al. 2013). In rats, maternal separation-induced ultrasonic vocalizations (USVs) may be akin to ‘distress’ vocalizations or crying in human infants (Zeskind et al. 2011). These distress vocalizations provide a unique biologically and socially relevant signal essential for early survival and development (Zeskind et al. 2011). Similarly, previous studies in mice and rats have used maternal separation-induced USVs as an early developmental measure of disease (Hodgson et al. 2008; Scattoni et al. 2009; Bowers et al. 2013; Brudzynski 2013). For example, Foxp2 has previously been shown to mediate sex differences in Fibroblast growth factor 17 KO mice, a putative model for schizophrenia has impaired social behavior as well as decreased maternal separation-induced USVs (Scearce-Levie et al. 2008). Moreover, VP plays a role in maternal separation-induced USVs. For instance, systemic administration of the V1b antagonist SSR149415 reduces maternal separation-induced vocalizations in rat pups (Iijima and Chaki 2005). In contrast, central VP administration also reduced maternal separation-induced USVs (Winslow and Insel 1993). Interestingly, VP administered with a V1a antagonist had no effect on USVs, whereas VP administered with a V2 antagonist produced a reduction in USVs suggesting a role of V1a receptors in mediating USVs (Winslow and Insel 1993). Furthermore, the V1a/V2 antagonist JNJ-17308616 reduces maternal separation-induced USVs in rats as well (Bleickardt et al. 2009). Since increased and decreased VP signaling can mediate reductions in USVs, there is likely an optimal range and straying too far from this range results in impaired social behavior.

The Brattleboro (BRAT) rat carries a naturally occurring mutation of the Long-Evans rat with a single base deletion in exon 2 of the arginine vasopressin gene. This mutation results in an altered VP protein precursor and, as a result, no active VP production. The lack of VP in BRAT rat leads to a number of phenotypes including a decrease in dopamine levels in the frontal cortex as well as upregulation of striatal dopamine-2 receptors (Shilling et al. 2006; Cilia et al. 2010). Furthermore, adult BRAT rats demonstrate many behavioral deficits consistent with schizophrenia, such as increased startle, impaired cognitive performance, and reduced social function with mixed results regarding hyperactivity and anxiety phenotypes. (Brito et al. 1981; Gash et al. 1982; Warren and Gash 1983; Williams et al. 1983; Burnard et al. 1985; Herman et al. 1986; Stoehr et al. 1993; Engelmann and Landgraf 1994; Ingram et al. 1998; Jentsch et al. 2003; Aarde and Jentsch 2006; Shilling et al. 2006; Mlynarik et al. 2007; Frank and Landgraf 2008; Feifel et al. 2009, 2011; Schank 2009; Zelena et al. 2009; Cilia et al. 2010). Interestingly, at postnatal days 7 and 10, BRAT rats are hyperactive and have social deficits, consistent with both prodromal symptoms in schizophrenia as well as early developmental abnormalities in autism spectrum disorders (Schank 2009).

In this study, we assess the extent to which BRAT rats display deficits in the vocal communicative domain. Additionally, we determined the effect of reduced VP on auditory ERPs, as this measure shows distinct profiles in schizophrenia (Turetsky et al. 2009, 2007; Gandal et al. 2010).

## Experimental Procedures

### Animals

BRAT rats were bred at the University of Pennsylvania from two heterozygous breeding pairs (RrrcHsdBlu:BRAT-*Avp*^*+/di*^) purchased from the Rat Resource & Research Center (RRRC, Columbia, MO). All subsequent rats were bred from heterozygous pairs in an Association for Assessment and Accreditation of Laboratory Animal Care-accredited animal facility and housed two to three per cage after weaning in a light- and temperature-controlled room. Animals used for EEG experiments were single housed after electrode implantation. 2House lights were on between 6 am and 6 pm and water and standard rodent chow were available ad libitum. Ultrasonic vocalization studies and ERP were conducted in separate cohorts of male homozygous knockout Brattleboro (BRAT) rats (*Avp*^*di/di*^) and their littermate wild-type (WT) controls (*Avp*^*+/+*^) at the University of Pennsylvania during the light phase between 9:00 am and 5:00 pm. Due to the water retention impairments associated with the BRAT rats, cages were changed on a daily basis. Genotypes were determined by sequencing DNA extracted from tail snips for the Avp gene according to protocol provided by the RRRC (RRRC). All protocols were performed in accordance with University Laboratory Animal Resources guidelines and were approved by the Institutional Animal Care and Use Committee at the University of Pennsylvania (Protocol #804371).

### Ultrasonic vocalizations

#### Maternal separation-induced ultrasonic vocalizations

Male WT and BRAT rat pups from the same litters were assessed for maternal separation-induced distress ultrasonic vocalizations (USVs) on postnatal days 2, 5, 9, and 12 (WT *n* = 14, BRAT *n* = 9). Pups were removed from their home cage and placed in an empty plastic container 7″(W) × 11″ (D) × 5″ (H) with ambient air temperature maintained between 21 and 22°C. The chamber was cleaned between animals using 70% ethanol and allowed to air dry prior to testing further pups. Vocalizations were recorded for 120 sec using an ultrasonic range detector placed 10 cm above the plastic container (Pettersson Electronik D940 Ultrasound Detector, Uppsala, Sweden). Vocalizations were recorded for 120 sec to minimize stress caused by maternal separation. The microphone was placed 10 cm above the pup and was interfaced with a Micro1401 data acquisition unit (CED) sampling at 200 kHz recorded through Spike2 software (CED). Data were imported into Matlab for analysis. Sonograms were generated using a fast Fourier transform (FFT) of length 512 points using a Hanning window and infinite impulse response filtering between 25 and 55 kHz. The frequency range was chosen to capture all visible vocalizations on the sonogram of each individual rat pup for all age ranges tested. Vocalization frequency, interval, duration, and power were recorded using a variation of Matlab scripts available as freeware from the laboratory of Dr. Tim Holy (Holy and Guo 2005; Ise and Ohta 2009). Power was calculated using Fast Fourier Transform (FFT) of each call between 25 and 55 kHz and averaging the power of each call together as described (Holy and Guo 2005).

#### Social interaction-induced ultrasonic vocalizations

Two previously unexposed male rats of the same genotype (Pairs, WT *n* = 10, BRAT *n* = 8) were simultaneously placed into the middle of a 32″(W) × 18″ (D) × 12″(H) plastic chamber without bedding and video was recorded for 5 min in low light. Simultaneously, USVs were recorded in a similar fashion as with maternal separation-induced USVs with the exception that the microphone was 30 cm from the base of the chamber. The chamber was cleaned with 70% ethanol between recordings. The video was scored for the amount of time the animals spent socially interacting and vocalizations were manually counted. A social interaction was considered to be any time the snout of one animal was in direct contact with the other animal. All statistics were performed with Statistica software package (StatSoft, Inc., Tulsa, OK) using repeated measures ANOVA. Post hoc analyses were performed with Fisher LSD with a significance threshold of *P* = 0.05.

### Event-related potentials

#### Electrode implantation

Rats between 300 and 400 g, (WT *n* = 11, BRAT *n* = 11) underwent stereotaxic implantation of two 3-channel electrodes (PlasticsOne Inc., Roanoke, VA) under isoflurane anesthesia. One electrode was placed in the prelimbic cortex (3.2 mm anterior, 1 mm lateral, and 4 mm ventral relative to bregma) and the other electrode was placed in the ipsilateral cerebellum which was used as a reference and ground (2 mm posterior, 2 mm lateral, and 2 mm ventral relative to lambda) in a similar fashion as that previously reported (Connolly et al. 2004, 2003; Maxwell et al. 2004; Ehrlichman et al. 2009). Since the recording and reference electrodes were located far apart from one another, activity recorded using this configuration extends far beyond the localized field generated within the prelimbic cortex, and, therefore reflects brain activity across a widespread area. Electrodes were placed in the prelimbic cortex because previous studies have shown prefrontal cortex impairments in schizophrenia (Edgar et al. 2012; Lisman 2012; Yoon et al. 2013). Histological verification of electrode placement was performed following the completion of experiments. Dental cement and ethyl cyanoacrylate (Elmers, Columbus, OH) were used to secure the electrode pedestal to the skull. Procedures were consistent with descriptions published elsewhere (Connolly et al. 2004, 2003; Siegel et al. 2005; Metzger et al. 2007).

#### Recording

After a minimum 1-week recovery time, ERPs were recorded during the presentation of an auditory task. Rats were transferred to a clean cage and cables were attached 20 min prior to recording. The auditory task consisted of a single click paradigm with presentation of a 9 kHz tone (10 ms, 85 dB) at a 4-sec interstimulus interval 200 times against 70 dB of background noise. EEG was recorded with a sampling rate of 1667 Hz and band-pass filtered between 1 and 500 Hz during collection. Stimuli were generated by Power 1401 hardware and Spike 6 software (Cambridge Electronic Design, Cambridge, UK) and were delivered through speakers attached to the cage top. All recordings were performed in a plastic cage 7″(W) × 11″ (D) × 5″ (H) with standard bedding, which was placed in a Faraday cage 15 min before stimulus onset.

#### Analysis

For each animal, individual trials were rejected for movement artifact defined by two times the root mean square of the amplitude per rat. Average waves were created from 1000-ms prestimulus to 1000-ms poststimulus time. The N40 component was defined as the maximum negative deflection between 25 and 60 ms. Power analysis was performed using EEGLAB (Schwartz Center for Computational Neuroscience). Event-related low gamma (30–80 Hz and 0–60 ms) and high gamma (80–120 Hz and 0–60 ms) power were calculated using Morlet wavelets in 116 logarithmically spaced frequency bins between 4 and 120 Hz, with wavelet cycle numbers ranging from 2 to 10. All statistics were performed with Statistica software package using repeated measures ANOVA. Post hoc analyses were performed with Fisher LSD with a significance threshold of *P* = 0.05.

## Results

### Social interaction

**Figure 1:**
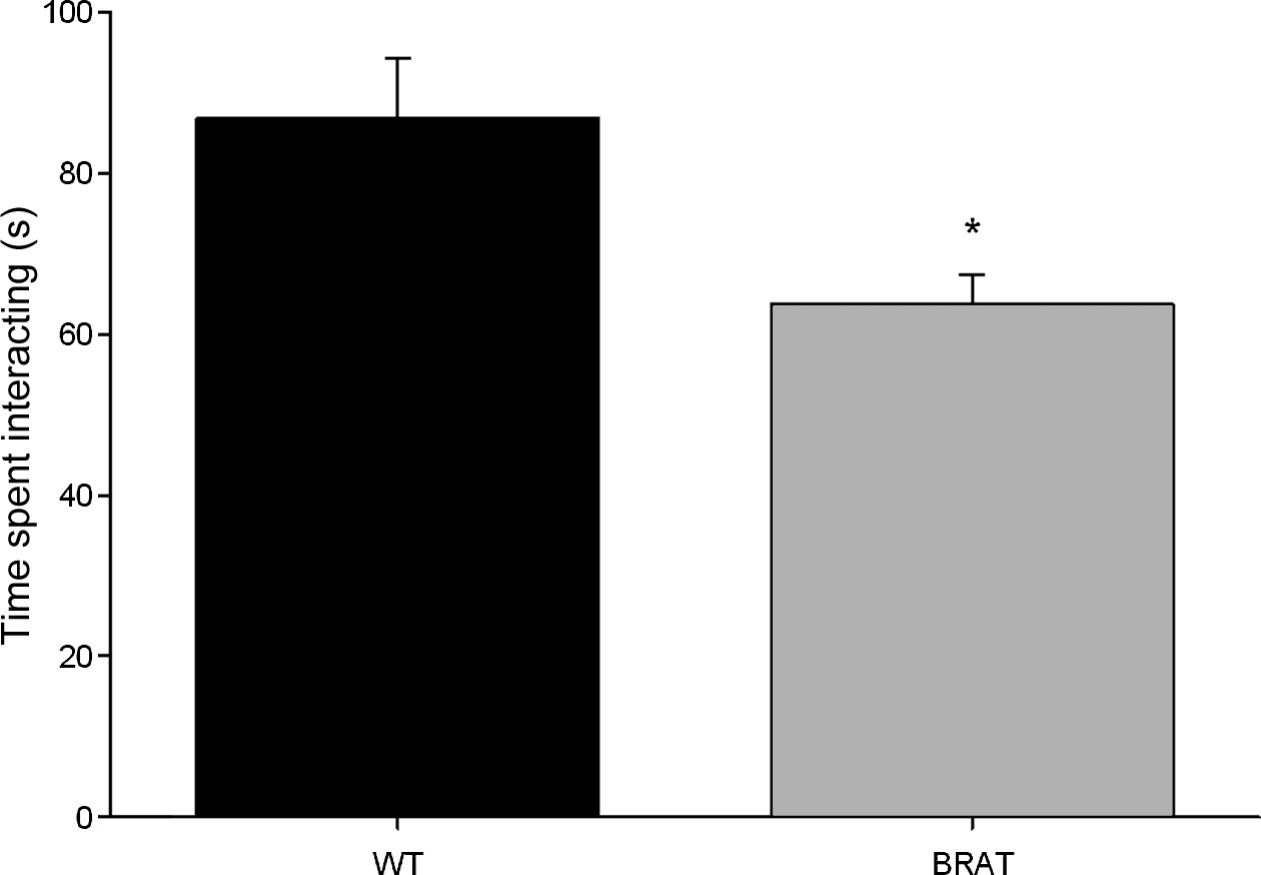
Social interaction was assessed for 5 min between two freely interacting male adult rats of the same genotype. BRAT rats spent less time in direct contact with each other compared to littermate controls (*P < 0.05).

Adult BRAT rats spent less time interacting with each other than WT littermate controls in the freely interacting social interaction paradigm (Fig. 1, *F*_1,16_ = 6.79, *P* = 0.019). These deficits are consistent with prior reports and suggest that animals bred at the University of Pennsylvania share phenotypic deficits with those that have been previously described (Feifel et al. 2009).

### Ultrasonic vocalizations

**Figure 2:**
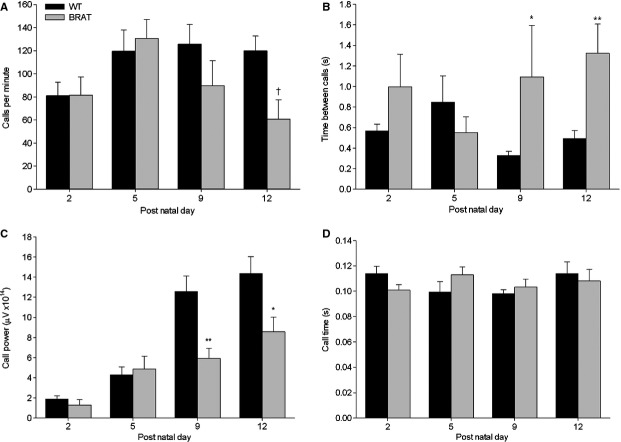
Maternal separation-induced distress ultrasonic vocalizations were measured in wild-type and BRAT rats during the first 12 days of life. USVs were analyzed for (A) number of calls, (B) intercall interval, (C) call power, and (D) call duration. There was a trend toward a reduction in call frequency in BRAT rats on postnatal day 12. There was also a significant increase in intercall interval that was accompanied by a reduction in call power on postnatal days 9 and 12. No change was observed in call duration (^†^*P* < 0.1, *P < 0.05, **P < 0.01).

USV recordings on days 2, 5, 9, and 12 after birth were analyzed for number of calls, intercall interval (time between calls), call power, and call duration. There was an overall increase in the number of calls through postnatal day 12 (Fig. 2A, *F*_3, 63_ = 3.39, *P* = 0.023). Post hoc analysis indicated a trend in the BRAT rats toward a reduction in the number of calls (Fig. 2A, *F*_3, 63_ = 2.38, *P* = 0.078). The intercall interval showed an overall increase among the BRAT rats (Fig. 2B, *F*_1, 19_ = 11.18, *P* = 0.0035). Post hoc analysis indicated that the intercall interval was significantly longer in BRAT rats on postnatal days 9 and 12 (MS = 0.54, df = 74.61 *P* = 0.016 and *P* = 0.0097, respectively). Although both genotypes showed an increase in power over time (*F*_3, 60_ = 30.21, *P* < 0.0001), the call power was increased to a lesser extent in Brat rats when compared to littermate controls (Fig. 2C, *F*_1, 20_ = 4.75, *P* = 0.041). Post hoc analysis showed that the power of the BRAT rats calls were specifically reduced on days 9 and 12 after birth (MS = 174E27, df = 72.61, *P* = 0.0091, *P* = 0.036). The duration of the calls was consistent across genotype and the testing days (Fig. 2D, *F*_3,60_ = 0.95, *P* = 0.42). There was no difference among adult BRAT rats and their littermate controls for the number of USVs emitted during a social interaction (*F*_1,10_ = 0.36, *P* = 0.56, data not shown).

### EEG/ERP

**Figure 3:**
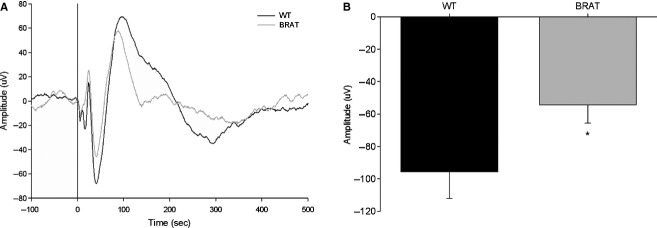
Event-related potential (ERP) profiles in wild-type and BRAT rats are shown. ERP components were extracted by averaging epochs resulting in (A) averaged ERP and (B) N40 amplitude. The BRAT rats have reductions in N40 amplitude (*P = 0.008).

**Figure 4:**
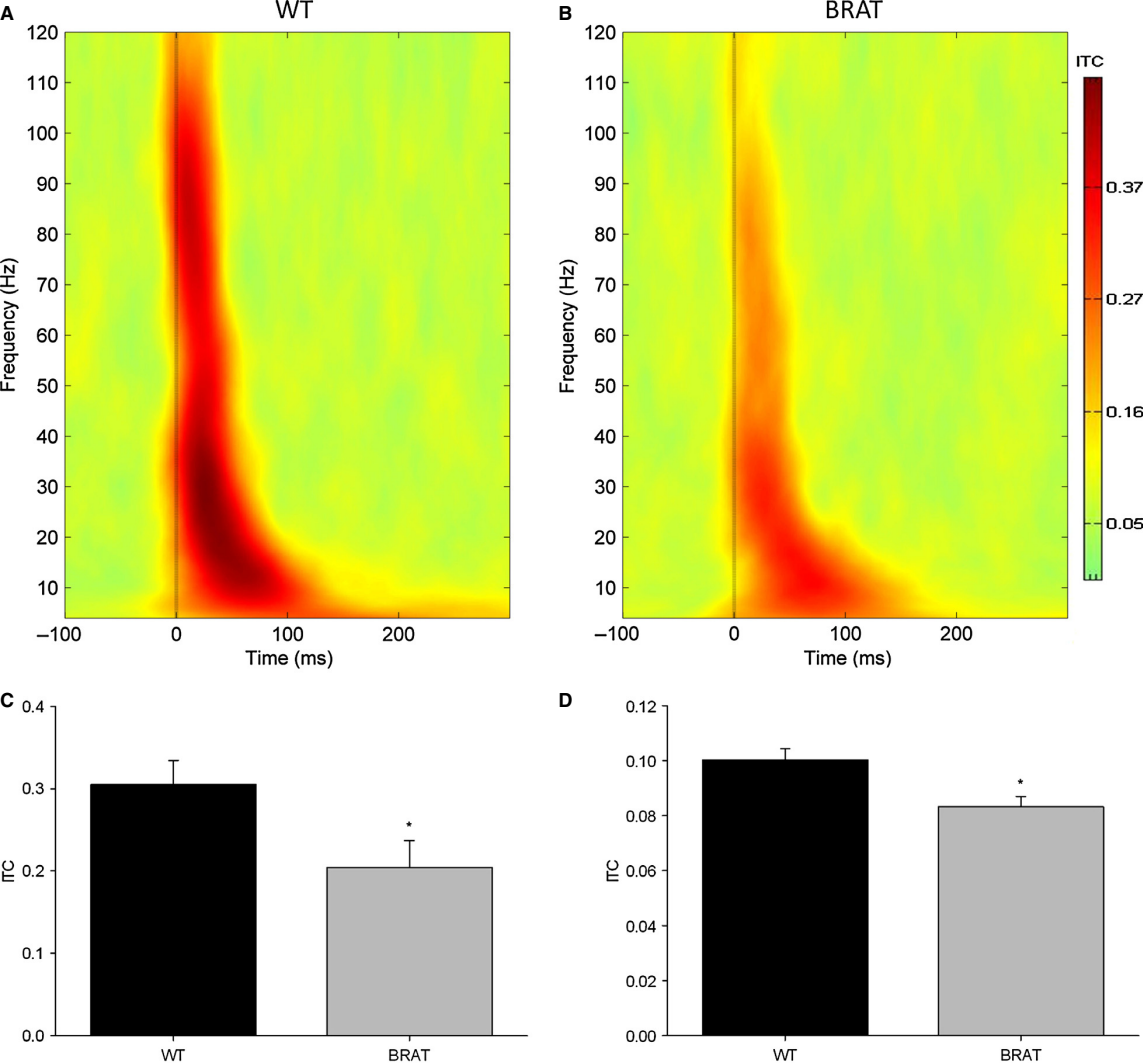
Time frequency analysis of the EEG phase locked to the auditory stimulus illustrated as a heat map for (A) wild-type and (B) BRAT rats. The averaged phase-locked frequency components are depicted for (C) low gamma ITC, and (D) high gamma ITC. The BRAT rats have reductions in low gamma and high gamma ITC (*P < 0.05).

WT and BRAT rat EEG and ERP components were recorded and analyzed during an auditory task. No significant differences in ERP component latency (P20, N40, and P80) were observed between BRAT rats and WT littermate controls (Table 1). However, a significant reduction in N40 amplitude was observed among BRAT rats (Fig. 3A,B, *F*_1, 20_ = 8.65, *P* = 0.008). Time spectrum analyses revealed a significant reduction in intertrial coherence (Fig. 4A,B, ITC) for both low (0–60 s, 30–80 Hz) (Fig. 4C, *F*_1, 20_ = 5.16, *P* = 0.034) and high (0–60 s 80–120 Hz) gamma (Fig. 4D, *F*_1, 20_ = 9.39, *P* = 0.006), suggesting that cross-trial synchronization/consistency of gamma oscillations is disrupted by removal of VP.

**Table 1:** Latencies and amplitudes of ERP component peaks.

	Latency			Peak		
		
	P20	N40	P80	P20	N40*	P80
WT	22.4 ± 1.6	45.7 ± 2.3	116.2 ± 8.9	22.1 ± 7.7	−106.1 ± 15.9	86.6 ± 15.0
BRAT	24.5 ± 1.1	45.05 ± 2.2	96.8 ± 9.9	32.5 ± 4.4	−54.3 ± 11.3	74.6 ± 18.4

*P = 0.008.

## Discussion

In this study, we report a reduction in neonatal USV during maternal separation as well as EEG oscillatory deficits in adult rats in BRAT rats that lack VP. The VP deficit-mediated reduction in auditory communication during early development (postnatal days 9 and 12) suggests that the effect of VP on social behavior is age dependent and emerges at time points corresponding to early postnatal period in humans (Zeskind et al. 2011). Previous data have shown that treatment with a V1b antagonist similarly causes reductions in maternal separation-induced vocalizations (Iijima and Chaki 2005). However, VP may also be interacting with other neurotransmitter systems. For example, the D1 agonist SKF81297 and the mGluR5 antagonist MPEP both produce similar reduction in maternal separation-induced vocalizations in rats (Iijima and Chaki 2005; Muller et al. 2009). In addition to the vocalization deficits, BRAT rats have abnormalities in locomotor activity and impairments in social behavior at postnatal days 7 and 10 (Schank 2009).

During adulthood, BRAT rats also have many behavioral deficits that are consistent with schizophrenia. Previous studies have shown that BRAT rats have deficits in prepulse inhibition of startle, social discrimination, and attentional engagement (Jentsch et al. 2003; Feifel et al. 2009, 2011). Additionally, we demonstrate that BRAT rats have reduced social interactions, consistent with previous studies and reduced social function in schizophrenia (Feifel et al. 2009). Interestingly, there were no quantitative deficits in the number of calls emitted among adult BRAT pairs during free social interaction. However, it is possible that adult BRAT rats may have subtle changes in the qualitative nature and types of calls rather than amount. Such changes could be informative regarding changes in prosody and inflection among people with schizophrenia (Jahshan et al. 2013). Therefore, future studies will assess such measures in BRAT rats. In addition to the behavioral deficits, we demonstrate a deficit in auditory ERPs similar to those seen in schizophrenia patients (Shin et al. 2010; Sharma et al. 2011; Swerdlow et al. 2012). BRAT rats have a reduction in low and high gamma intertrial coherence, indicating a reduction in the phase coherence and consistency of response to stimuli in the brain. Furthermore, previous studies conducted in mouse models have shown that NMDA hypofunction plays a critical role in the determination of ITC (Saunders et al. 2012a,b).

Pharmacological and parametric manipulations have suggested a close correspondence between the rodent N40 and human N100 (Metzger et al. 2007; Amann et al. 2008; Swerdlow et al. 2012). For example, pharmacological manipulations using dopamine agonists and nicotinic cholinergic agonists in rodents produce alterations in N40 that closely overlap with the effects such drugs have on the human N100 (Siegel et al. 2005; Maxwell et al. 2006; Kanes et al. 2007; Metzger et al. 2007; Amann et al. 2010; Halene and Siegel 2008; Rudnick et al. 2009, 2010; Cao et al. 2012; Featherstone et al. 2012). As such, it is likely that the reduction in N40 amplitude observed here in BRAT rats is analogous to the N100 reductions observed in schizophrenia patients. One potential explanation for the N40 deficit is an increase in the variability of the timing of the peak as opposed to a reduction in the amplitude of the peak (Jansen et al. 2010). In fact, variability of the time point at which the maximum deflection occurs between individual trials of the N100 peak may account for the reduction in average peak amplitude in schizophrenia patients (Jansen et al. 2010). There was no change in the N40 peak latency, an endophenotype typically associated with autism spectrum disorders (Gandal et al. 2010). Altogether, the EEG data suggest a VP-driven modulation of dopaminergic and glutaminergic tone in the BRAT rats.

The neonatal developmental deficits seen in BRAT rats are incongruous with the onset of prodromal deficits seen in many schizophrenia patients, which are primarily manifested during adolescence (Mees et al. 2011). It is possible to interpret these findings as more similar to autism spectrum disorders, which typically is diagnosed during infancy (Doyle and McDougle 2012). However, the BRAT rats exhibit deficits during adulthood such as the EEG deficits, which are not consistent with autism spectrum disorders. It remains unclear if the ERP deficits in BRAT rats develop during adolescence or are already present during early development but further studies are recommended to address this concern.

## Conclusion

The role of VP in the etiology of schizophrenia remains unclear. One hypothesis posits that there is an increase in dopamine system function among BRAT rats. Previous data have shown decreased dopamine levels in the frontal cortex as well as upregulation of striatal dopamine-2 receptors in BRAT rats (Shilling et al. 2006; Cilia et al. 2010). The dopamine hypofunction in the frontal cortex accompanied by the dopamine hyperfunction in the striatum are consistent with the dopamine hypothesis for schizophrenia (Howes and Kapur 2009). Additionally, BRAT rats have reductions in PPI and elevations in startle response. These are all effects that are elicited by dopamine agonist administration (Talalaenko et al. 2006). However, the EEG deficits in ITC reported here cannot be explained with the dopamine hypothesis, suggest the involvement of another system or systems. Interestingly, VP also acts as a neurotransmitter in the amygdala and lateral septum; structures that are implicated in social and anxiety behaviors. Glutamate hypofunction can model the EEG deficits elicited in this study and social impairments previously reported. Therefore, it is conceivable that VP, acting as a neurotransmitter and neuromodulator, is able to model multiple endophenotypes of schizophrenia. Augmentation of the VP system may be useful as a novel therapeutic for schizophrenia. Consistent with this idea, a small study using a VP agonist as a treatment for negative symptoms and memory in schizophrenia demonstrated an improvement in negative symptoms and a trend toward improvement in memory (Brambilla et al. 1989). In conclusion, augmentation of the VP system may provide a novel treatment for the negative symptoms of schizophrenia and BRAT rats could provide valuable insight into the mechanisms in which these manipulations would have their effect.
